# Periodontal regeneration using platelet‐rich fibrin. Furcation defects: A systematic review with meta‐analysis

**DOI:** 10.1111/prd.12583

**Published:** 2024-09-26

**Authors:** Richard J. Miron, Vittorio Moraschini, Nathan E. Estrin, Jamil Awad Shibli, Raluca Cosgarea, Karin Jepsen, Pia‐Merete Jervøe‐Storm, Anton Sculean, Søren Jepsen

**Affiliations:** ^1^ Department of Periodontology University of Bern Bern Switzerland; ^2^ Department of Oral Surgery Fluminense Federal University Niterói, Rio de Janeiro Brazil; ^3^ School of Dental Medicine Lake Erie College of Osteopathic Medicine Bradenton, Florida USA; ^4^ Department of Implant Dentistry, School of Dentistry Guarulhos University Guarulhos, São Paulo Brazil; ^5^ Department of Periodontology, Operative and Preventive Dentistry University of Bonn Bonn Germany; ^6^ Clinic of Periodontology and Peri‐implant Diseases University of Marburg Marburg Germany; ^7^ Faculty of Dentistry University Iuliu Hatieganu Cluj‐Napoca Romania

**Keywords:** advanced‐PRF, horizontal centrifugation, furcation defect, leukocyte and platelet‐rich fibrin, L‐PRF, meta‐analysis, periodontal regeneration, periodontitis, systematic review

## Abstract

The objective of the study was to compare the treatment outcomes of periodontal furcation defects by using platelet‐rich fibrin (PRF) with other commonly utilized modalities. The eligibility criteria comprised randomized controlled trials (RCTs) comparing the clinical outcomes of PRF with those of other modalities for the treatment of furcation defects. Studies were classified into 11 categories in 3 different groups as follows: Group I (addition of PRF): (1) open flap debridement (OFD) alone versus OFD/PRF, (2) OFD/bone graft (OFD/BG) versus OFD/BG/PRF; Group II (comparative studies to PRF): (3) OFD/BG versus OFD/PRF, (4) OFD/collagen membrane versus OFD/PRF, (5) OFD/PRP versus OFD/PRF, (6) OFD/rhBMP2 versus OFD/PRF; and Group III (addition of biomaterial/biomolecule to PRF): OFD/PRF versus … (7) OFD/PRF/BG, (8) OFD/PRF/amniotic membrane (AM), (9) OFD/PRF/metformin, (10) OFD/PRF/bisphosphonates, (11) OFD/PRF/statins. Weighted means and forest plots were calculated for the reduction of probing pocket depth (PPD), gain of vertical and horizontal clinical attachment levels (VCAL and HCAL), gain in vertical and horizontal bone levels (VBL, HBL), and radiographic bone fill (RBF). From 45 articles identified, 21 RCTs reporting on class II furcations were included. The use of OFD/PRF and OFD/BG/PRF statistically significantly reduced PPD and improved VCAL and HCAL when compared to OFD or OFD/BG, respectively. The comparison between OFD/PRF alone versus OFD/BG, OFD/CM, OFD/PRP, or OFD/rhBMP2 led to similar outcomes for all investigated parameters, including a reduction in PPD, VCAL/HCAL gain, and RBF. The additional incorporation of a BG to OFD/PRF only mildly improved outcomes, whereas the addition of AM improved clinical outcomes. The addition of small biomolecules such as metformin, bisphosphonates, or statins all led to significant improvements in PPD, VCAL, and HCAL when compared to OFD/PRF alone. Noteworthy, a very high heterogeneity was found in the investigated studies. The use of PRF significantly improved clinical outcomes in class II furcation defects when compared to OFD alone, with similar levels being observed between OFD/PRF and/or OFD/BG, OFD/CM, OFD/PRP, or OFD/rhBMP2. Future research geared toward better understanding potential ways to enhance the regenerative properties of PRF with various small biomolecules may prove valuable for future clinical applications. Future histological research investigating PRF in human furcation defects is largely needed. The use of PRF in conjunction with OFD statistically significantly improved PPD, VCAL, and HCAL values, yielding comparable outcomes to commonly used biomaterials. The combination of PRF to bone grafts or the addition of small biomolecules may offer additional clinical benefits, thus warranting future investigation.

## 1 INTRODUCTION

Molars with classes II and III furcation defects are recognized as complexity factors for determining the stage of periodontitis in the new AAP/EFP classification of periodontal diseases.^1^, ^2^ This is due to the fact that they exhibit a higher rate of tooth loss than molars without furcation involvement^3^ and provide a significant challenge for the clinician to accomplish a successful treatment.^4^ Although access flap surgery (open flap debridement) and nonsurgical therapies have had mixed results,^5^, ^6^, ^7^, ^8^ regenerative periodontal surgery has been shown to provide better results with furcation improvement, especially in cases of class II furcations.^7^, ^9^, ^10^, ^11^, ^12^ The S3‐level clinical practice guideline (CPG) for the treatment of periodontitis recommends the use of bone grafts, resorbable membranes, and enamel matrix derivatives for furcation regeneration due to their proven clinical effectiveness as shown by randomized studies.^7^, ^13^ Despite the encouraging potential of autogenous platelet concentrates, the discussion of their possible application for these indications was not possible due to the insufficient evidence included in this CPG.^14^

More recently, a number of systematic reviews including meta‐analyses have assessed the benefits of using platelet‐rich fibrin (PRF) to treat periodontal furcation defects and have demonstrated their usefulness under these therapeutic settings.^15^, ^16^, ^17^, ^18^

This systematic review with meta‐analysis aims to expand the database and evaluate the most up‐to‐date evidence on the efficacy of PRF in treating furcation defects, compared to, and in combination with, alternative treatment options such as membranes, bone grafts, and other biomolecules commonly used for periodontal regeneration.

## 2 MATERIALS AND METHODS

### 2.1 Protocol

This SR adhered to the PRISMA standards' guidelines.^19^ This SR's protocol was built using the PRISMA‐P framework.^20^ There were no deviations from the initial protocol. This SR's protocol was registered with the INPLASY database under the identifier 2023100045.

### 2.2 Focused question

Three specific issues regarding the impact of PRF in treating class II furcation defects were taken into consideration for this SR:

In patients/teeth affected by periodontitis‐related class II furcation defects, what is the efficacy of the use of PRF in regenerative periodontal surgery in terms of furcation improvement (outcome variables: reduction in probing pocket depth (PPD) and gain in vertical and horizontal clinical attachment level (VCAL) and gain in vertical and horizontal bone level (HBL, VBL), horizontal bone level (HBL) and radiographic bone fill (RBF).) compared to:

1. Therapeutic modalities with/without PRF (FQ‐1).
2. Therapeutic modalities in comparison to PRF (FQ‐2).
3. Therapeutic modalities of PRF with the addition of biomaterials/biomolecules (FQ‐3).

### 2.3 Eligibility criteria and study selection process

The following PICOS approach served as the foundation for the inclusion criterion.^21^ Two independent reviewing authors, R.J.M. and N.E.E., carried out the search and screening procedure, starting with the examination of abstracts and titles. After that, complete papers were chosen for examination and compared to the requirements for data extraction. The reviewing authors carefully discussed and worked out any disagreements. Only research that fulfilled the specified requirements was included:

- Population: Individuals in good overall health who have periodontal class II furcation involvement.
- Intervention: Surgical treatment of furcation defects through the use of PRF alone or in combination with other biomaterials with a follow‐up period of at least 6 months.
- Comparison: PRF versus open flap debridement (OFD) alone or in combination with other biomaterials.
- Outcomes: *Primary* – reduction in probing pocket depth (PPD) and gain in vertical and horizontal clinical attachment level (VCAL and HCAL). *Secondary* – Gain in vertical and horizontal bone levels (VBL and HBL), radiographic bone fill (RBF), and furcation improvement (complete closure/conversion into class I).
- Study design: randomized controlled trials (RCTs) with a minimum of 10 patients.

### 2.4 Search strategy

**FIGURE 1:**
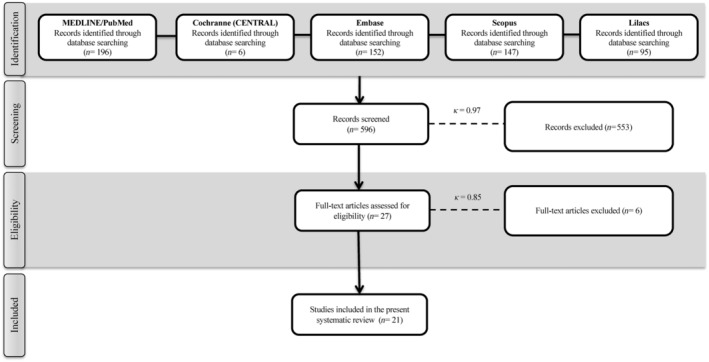
Search strategy.

PubMed/MEDLINE, the Cochrane Central Register of Controlled Trials, Scopus, Embase, and Lilacs were used to search for articles that were published before October 2023 without other restrictions regarding date or language. A Literature Report was used to conduct a gray literature search^22^ and additionally OpenGrey^23^ databases was also carried out. Additional studies that may be included were found by evaluating (or cross‐referencing) the study reference lists. The search strategy is described in Figure 1. The research excluded case reports, animal studies, retrospective clinical studies, and follow‐ups of less than 6 months.

### 2.5 Data synthesis

The research data were extracted in duplicate by R.J.M., N.E.E., P.‐M.J.‐S., and S.J.; they were then carefully examined by V.M. When available, the following data were extracted from the included studies: Researchers, authors, number of smokers, gender, age range, subjects, surgical technique, number of treated furcation defects, follow‐up, centrifugation parameters, amount of blood collected, centrifugation system, kind of bone defects, mean difference (MD) in PPD, VCAL, HCAL, VBL, HBL, RBF.

### 2.6 Assessments of the risk of bias

Two review authors (P‐M.J‐S. and S.J.) evaluated the methodological quality of the included studies primarily based on the risk of bias components that have been shown to impact research findings, such as the examiners' blinding, allocation concealment, and randomization methods. The risk of bias was done in duplicate. Assessments were also conducted regarding possible challenges to validity, selective result reporting, and the completeness of outcome reporting. The risk of bias was considered in sensitivity analyses to evaluate the results' robustness, but it was not used to exclude research that met review requirements.

The Cochrane Handbook for Systematic Reviews of Interventions' RoB 2 tool was used in this review.^24^, ^25^ It was used to examine the possibility of bias in RCTs. Every research article was examined in five areas: risk of bias arising from the randomization process, risk of bias due to deviations from the intended interventions, missing outcome data, risk of bias in the measurement of the outcome, and risk of bias in the selection of the reported research. Based on responses to the signaling questions, an algorithm generated a recommendation on the likelihood of bias resulting from each area. The recommendation may indicate “some concerns,” “high‐,” or “low‐”risk of bias. A study was considered “low” risk if every one of the five study areas was deemed low risk; “some concerns,” if it is determined that the research raises some issues in at least one category; and “high” risk, when at least one domain deems the research to be high risk. Since the clinician executing different surgical procedures cannot be blinded, we did not rate the surgeon's performance bias.

### 2.7 Statistical analysis

Review Manager Software (version 5.2.8, Copenhagen, Denmark, 2014) was used to perform a meta‐analysis after the continuous variables (PPD, VCAL, and HCAL) from the included studies were split into groups and subgroups.

The effects were assessed using the mean difference with confidence interval (CI) of 95%. It was decided to use the generic variation technique. Chi‐square tests were used to assess the heterogeneity, with values ≤25% indicating low heterogeneity, values >25% but ≤50% indicating moderate heterogeneity, and values >50% indicating high heterogeneity.^26^ For the analyses, the random effect model was chosen due to the variation in available evidence (e.g., populations, follow‐up times, and settings). The statistical significance level used for the meta‐analysis effect was *p* < 0.05.

### 2.8 Risk of bias across studies

The many forms of reporting bias that could have existed in this study were considered.

If there were more than 10 studies included in a meta‐analysis, a funnel plot to detect possible publication bias should be created, and the Egger's and Begg's tests applied.^27^ However, an asymmetrical funnel plot may be due to other factors. In this review, there were no single comparisons including more than 10 studies.

## 3 RESULTS

### 3.1 Literature search

The initial search produced 196 titles from the MEDLINE/PubMed database, 6 from Cochrane (CENTRAL), 152 from Embase, 147 from Scopus, and 95 from Lilacs. The first evaluation of titles and abstracts excluded 575 articles that did not adhere to the eligibility criteria. Therefore, 21 studies on furcation defects^28^, ^29^, ^30^, ^31^, ^32^, ^33^, ^34^, ^35^, ^36^, ^37^, ^38^, ^39^, ^40^, ^41^, ^42^, ^43^, ^44^, ^45^, ^46^, ^47^, ^48^ published between 2011 and 2023 met the eligibility criteria and were included in this SR. Of the 21 RCTs, the most highly researched centrifugation system utilized in 10 of 21 studies (48% of studies) was the Remi centrifuge, whereas the Systonic Lab and Scientific Instruments, IntraSpin, Labtech‐Centifuge, and the Orthophos XG 3D/Ceph, Sirona Dental Systems GmbH system were each utilized in 1 of the 21 studies (5% of studies each). Four of the 21 studies did not report the centrifuge utilized (19%). Of the 21 studies, 12 utilized a 3000 rpm for 10 min protocol (57% of studies), 2 studies utilized a 400 relative centrifugal force (RCF) for 10 min protocol (10% of studies), whereas each of the remaining protocols were utilized in 1 of 21 studies including a 400 RCF × 12‐min, 3000 rpm × 12‐min, 700 rpm × 3‐min, or 2700 rpm × 12‐min, and a 2700 rpm × 10‐min protocol (5% each). No studies included smokers into their study.

### 3.2 Study characteristics

The included studies analyzed 786 research participants. In addition to OFD alone, the effect of PRF was compared to other groups of biomaterials (BGs, CM, PRP, rhBMP2, AM, metformin, bisphosphonates, and statins). The mean follow‐up period of the studies was 9.42 months. The data extracted from each included study are presented in Table 1.

**TABLE 1:** Main characteristics of the 21 RCTs included in this study investigating furcation defects treated with PRF.

Authors (year)	Study design follow‐up	No. of participants	Groups	Furcation degree	Smokers (no, yes)	Conclusions
		Gender		Location		
		Mean age		Additional info		
**GROUP 1: Therapeutic modalities with/without PRF**						
*OFD vs. PRF*						
Sharma and Pradeep (2011)^28^	RCT (split‐mouth) 9 months	18 ♂10/♀8 34	C: 18, OFD T: 18, OFD + PRF	II Buccal Mandibular No recession	No	Significant improvement with autologous PRF implies its role as a regenerative material in the treatment of FD.
Bajaj et al. (2013)^29^	RCT (parallel) 9 months	42 ♂22/♀20 39	C: 23, OFD T:24, OFD + PRF	II Buccal Mandibular No recession	No	The use of autologous PRF was effective in the treatment of furcation defects with uneventful healing of sites.
Siddiqui et al. (2016)^30^	RCT (parallel) 6 months	31 ♂24/♀7 40.9	C: 15, OFD T:15, OFD + PRF	II Buccal Mandibular Gingiva coronal or at roof of furcation	No	There was statistically significant improvement at 6 months follow‐up from baseline values
Kanoriya et al. (2017)^31^	RCT (parallel) 9 months	47 ♂23/♀24 39	C: 24, OFD T:23, OFD + PRF	II Buccal Mandibular No extensive recession	No	FD treatment with PRF results in significant therapeutic outcomes when compared with OFD alone
Agarwal et al. (2019)^32^	RCT (parallel) 9 months	46 (for all 3 groups) ♂20/♀26 48	C: 20, OFD T: 20, OFD + PRF	II Buccal Mandibular	No	PRF was significantly advantageous for the management of mandibular degree II furcation defects
*BG vs. BG + PRF*						
Lohi et al. (2017)^33^	RCT (parallel) 6 months	14 ♂10/♀4 42.3	C: 8, OFD + BG T: 10, OFD + BG + PRF	II Buccal/lingual Mandibular Gingiva coronal or at roof of furcation	No	Adjunctive use of PRF with bone graft may be a more effective treatment
Rani et al. (2018)^34^	RCT (parallel) 6 months	20 NR NR	C: 10, OFD + BTCP T: 10, OFD + BTCP + PRF	II NR Mandibular	No	Adjunctive use of PRF with bone graft may be a more effective treatment
Basireddy et al. (2019)^35^	RCT (split‐mouth) 6 months	14 NR 30–50	C: 14, OFD + DFDBA T: 14, OFD + DFDBA + PRF	II Buccal/lingual Mandibular	No	PRF seems to favor soft‐tissue healing but has no additional benefit in bone regeneration when used in combination with DFDBA
Dambhare et al. (2019)^36^	RCT (parallel) 12 months	24 NR 40	C: OFD + HA and β‐TCP T: OFD + HA and β‐TCP + PRF	II Buccal/lingual Mandibular Proximal bone coronal to Interradicular bone	No	The treatment with PRF in combination with HA and β‐TCP group resulted in a significantly higher CAL gain, PPD and HPD reduction
Serroni et al. (2022)^37^	RCT (parallel) 6 months	28 ♂14/♀14 54	C: 14, OFD + AB T: 14, OFD + AB + PRF	II Buccal/lingual Mandibular	No	The addition of L‐PRF to ABG produces a significantly greater HCAL gain and PD reduction as compared with OFD + ABG treatment in mandibular degree II furcation involvements
Nair et al. (2022)^38^	RCT (parallel) 9 months	24 ♂12/♀12 35.3	C: 12, OFD + nano‐HA T: 12, OFD + nano‐HA + i‐PRF	II Buccal Mandibular	No	The results showed increased improvement in clinical conditions with better results in the i‐PRF + nano‐HA.
**Group 2: Therapeutic modalities in comparison to PRF**						
*BG vs. PRF*						
Biswas et al. (2016)^39^	RCT (parallel) 6 months	15 ♂10/♀5 38	C: 10, OFD + BG T: 10, OFD + PRF	II NR Mandibular	No	PRF seems to favor soft‐tissue healing but has no additional benefit in bone regeneration when compared to BG
Siddiqui et al. (2016)^30^	RCT (parallel) 6 months	30 ♂23/♀7 40.9	C: 15, OFD + BG T:15, OFD + PRF	II Buccal Mandibular Gingiva coronal or at roof of furcation	No	For both test and control groups, there was statistically significant improvement at 6 months follow‐up from baseline values
Asimuddin et al. (2017)^40^	RCT (parallel) 9 months	22 ♂14/♀8 30–50	C: 11, OFD + DFDBA + CM T: 11, OFD + PRF	II Buccal Mandibular	No	The intergroup comparison for PI, PD, RHCAL, GML was statistically not significant.
*CM vs. PRF*						
Mehta et al. (2018)^41^	RCT (split‐mouth) 6 months	18 NR NR	C: 18, OFD + DFDBA + CM T: 18, OFD + DFDBA + PRF	II Buccal/lingual Mand./max	No	Both groups showed statistically significant outcomes in intragroup comparison from baseline to 3 and 6 months. However, no statistical difference between PRF and collagen membrane groups were reported
*PRP vs. PRF*						
Bajaj et al. (2013)^29^	RCT (parallel) 9 months	42 ♂22/♀20 39	C: 23, OFD + PRP T:24, OFD + PRF	II Buccal Mandibular No recession	No	The use of autologous PRF or PRP were both effective in the treatment of FD with uneventful healing of sites
*rhBMP2 vs. PRF*						
Sneha et al. (2021)^42^	RCT (parallel) 6 months	32 NR 20–55	C: 16, OFD + collagen sponge + rhBMP2 T: 16, OFD + PRF	II NR NR	No	RhBMP‐2 in absorbable collagen sponge was effective in increasing the bone fill in Grade II furcation defects when compared to PRF alone (*p* = 0.05). In relation to clinical parameters, both the groups showed no statistical significance between them
**GROUP 3: Therapeutic modalities of PRF with addition of biomaterials/biomolecules**						
*PRF vs. PRF + BG*						
Agarwal et al. (2019)^32^	RCT (parallel) 9 months	46 (for all 3 groups) ♂20/♀26 48	C: 20, OFD + PRF T: 20, OFD + PRF + DFDBA	II Buccal Mandibular	No	PRF + DFDBA + OFD has significantly greater benefits than PRF + OFD in VBF
*PRF vs. PRF + AM*						
Kaur and Bathla (2018)^43^	RCT (split‐mouth) 6 months	15 ♂8/♀7 36.1	C: 15, OFD + PRF T: 15, OFD + PRF + AM	II Buccal Mandibular	No	Within the limitation of this study, there was greater pocket reduction, attachment level gain, and bone fill at sites treated with PRF and amnion membrane as compared to PRF alone
*PRF vs. PRF + metformin*						
Sharma et al. (2017)^44^	RCT (parallel) 6 months	22 ♂12/♀10 41	C: 15, OFD + PRF T: 15, OFD + PRF + MF	II Buccal Mandibular No recession	No	PRF when combined with a potential osteogenic agent like MF can provide a better therapeutic benefit to a furcation involved tooth
Swami et al. (2022)^45^	RCT (split‐mouth) 12 months	21 ♂10/♀11 43.3	C: 21, OFD + PRF T: 21, OFD + PRF + MF	II Buccal Mand./max.	No	Better clinical and radiographic findings in terms of reduction in PD, HPD, CAL gain, and significant reduction in DV in 1%MF + PRF
Dhande et al. (2023)^46^	RCT (split‐mouth) 6 months	23 NR 33–58	C: 23, OFD + PRF T: 23, OFD + PRF + MF	II Buccal Mand./max.	No	In Grade II furcation defects the combination therapy of 1% melatonin + PRF shows a statistically significant degree of bone fill within the periodontal tissues and also better results in terms of decrease in PPD, HPD, and a greater CAL gain
*PRF vs. PRF+ bisphosphonates*						
Kanoriya et al. (2017)^31^	RCT (parallel) 9 months	48 ♂25/♀23 38	C:23, OFD + PRF T2: 25, OFD + PRF + 1% ALN	II Buccal Mandibular No extensive recession	No	FD treatment with PRF combined with 1% ALN gel results in significant therapeutic outcomes when compared with PRF and OFD alone
Wanikar et al. (2019)^47^	RCT (split‐mouth) 6 months	20 ♂6/♀14 48	C: 20, OFD + PRF T: 20, OFD + PRF + 1%ALN	II Buccal Mand./max.	No	PRF + ALN treated defects exhibited better clinical and radiographic outcomes suggestive of enhanced periodontal regeneration when compared to PRF alone treated sites
*PRF vs. PRF + statins*						
Pradeep et al. (2016)^48^	RCT (parallel) 9 months	110 ♂60/♀50 (over 3 groups) 25–55	C0: 37, OFD C:36 OFD + HA + PRF T: 37, OFD + HA PRF + 1.2% RSV	II Buccal Mandibular No recession	No	OFD with RSV (1.2%) and PRF results in significantly greater periodontal benefits compared with OFD alone

Authors (year)	Mean difference in PPD between baseline and final follow‐up (mm), PPD = probing pocket depth	Mean difference in vCAL between baseline and final follow‐up (mm), vCAL = vertical clinical attachment level	Mean difference in hCAL (mm) between baseline and final follow‐up (mm), hCAL = horizontal clinical attachment level	Mean difference in vRBF between baseline and final follow‐up (mm), vRBF = vertical radiographic bone fill	Mean difference in vBF between baseline and final follow‐up (mm), vBF = vertical bone fill	Mean difference in hBF between baseline and final follow‐up (mm), hBF = horizontal bone fill
**GROUP 1: Therapeutic modalities with/without PRF**						
*OFD (C) vs. PRF (T)*						
Sharma and Pradeep (2011)	2.89 ± 0.68 (C) 4.06 ± 0.42 (T)	1.28 ± 0.46 (C) 2.33 ± 0.49 (T)	1.89 ± 0.76 (C) 2.67 ± 0.59 (T)	0.62 ± 0.22 (C) 2.01 ± 0.16 (T)		
Bajaj et al. (2013)	1.58 ± 1.02 (C) 4.29 ± 1.04 (T)	1.37 ± 0.58 (C) 2.87 ± 0.85 (T)	1.08 ± 0.50 (C) 2.75 ± 0.94 (T)	0.11 ± 0.03 (C) 1.85 ± 0.49 (T)		
Siddiqui et al. (2016)	1.03 ± 0.67 (C) 2.27 ± 1.10 (T1)	0.93 ± 0.46 (C) 2.40 ± 0.91 (T1)	0.73 ± 0.46 (C) 2.4 ± 1.06 (T1)		0.73 ± 0.46 (C) 1.93 ± 0.59 (T1)	1.07 ± 0.46(C) 2.13 ± 0.52 (T1)
Kanoriya et al. (2017)	2.41 ± 0.77 (C) 3.69 ± 0.76 (T)	2.33 ± 0.48 (C) 3.39 ± 0.49 (T)	2.04 ± 0.35 (C) 2.86 ± 0.06 (T)	0.52 ± 0.19 (C) 2.59 ± 0.32 (T)		
Agarwal et al. (2019)	1.50 ± 0.76 (C) 3.80 ± 0.77 (T)	1.35 ± 0.49 (C) 3.55 ± 1.05 (T)			0.45 ± 0.51 (C) 1.60 ± 0.88 (T)	0.20 ± 0.52(C) 1.40 ± 0.50(T)
*BG (C) vs. BG + PRF (T)*						
**Authors (year)**	**Mean difference in PPD between baseline and final follow‐up (mm)**	**Mean difference in vCAL between baseline and final follow‐up (mm)**	**Mean difference in hCAL (mm) between baseline and final follow‐up (mm)**	**Mean difference in vRBF between baseline and final follow‐up (mm)**	**Mean difference in vBF between baseline and final follow‐up (mm)**	**Mean difference in hBF between baseline and final follow‐up (mm)**
Lohi et al. (2017)	2.40 ± 0.52 (C) 3.37 ± 1.06 (T)	1.90 ± 0.57 (C) 3.00 ± 0.93 (T)	1.40 + 0.84 (C) 2.50 + 0.54 (T)		0.60 ± 0.70 (C) 1.38 ± 0.52 (T)	1.10 ± 0.88(C) 2.00 ± 0.76(T)
Rani et al. (2018)	3.50 ± 2.27 (C) 2.80 ± 1.93 (T)	2.80 ± 1.40 (C) 3.00 ± 1.49 (T)	3.70 ± 0.67 (C) 4.00 ± 0.88 (T)		3.50 ± 2.12 (C) 3.70 ± 1.57 (T)	
Basireddy et al. (2019)	2.36 ± 0.50 (C) 2.50 ± 0.52 (T)	1.79 ± 0.80 (C) 2.36 ± 0.50 (T)	1.50 ± 1.09 (C) 4.57 ± 1.70 (T)		1.01 ± 0. 66 (C) 1.19 ± 0.72 (T)	2.60 ± 0.81(C) 1.79 ± 1.43 (T)
Dambhare et al. (2019)	0.50 ± 0.52 (C) 2.00 ± 0.73 (T)	2.00 ± 0.85 (C) 3.33 ± 0.83 (T)	1.75 ± 1.21 (C) 3.33 ± 0.83 (T)			
Serroni et al. (2022)	2.15 ± 0.17 (C) 2.52 ± 0.17 (T)	1.99 ± 0.28 (C) 2.14 ± 0.28 (T)	1.61 ± 0.18 (C) 2.30 ± 0.18 (T)	1.72 ± 0.26 (C) 1.76 ± 0.25 (T)		
Nair et al. (2022)	3.40 ± 1.08 (C) 4.70 ± 0.77 (T)	2.50 ± 1.64 (C) 4.10 ± 0.75 (T)	1.70 ± 1.13 (C) 3.34 ± 1.10 (T)			
**Group 2: Therapeutic modalities in comparison to PRF**						
*BG (C)* vs. *PRF (T)*						
**Authors (year)**	**Mean difference in PPD between baseline and final follow‐up (mm)**	**Mean difference in vCAL between baseline and final follow‐up (mm)**	**Mean difference in hCAL between baseline and final follow‐up (mm)**	**Mean difference in vRBF between baseline and final follow‐up (mm)**	**Mean difference in vBF between baseline and final follow‐up (mm)**	**Mean difference in hBF between baseline and final follow‐up (mm)**
Biswas et al. (2016)	3.10 ± 0.62 (C) 3.70 ± 0.69 (T)	2.90 ± 0.86 (C) 3.70 ± 0.59 (T)				2.10 ± 0.63 (C) 2.30 ± 0.72 (T)
Siddiqui et al. (2016)	2.47 ± 1.51 (C) 2.27 ± 1.10 (T)	2.53 ± 0.83 (C) 2.40 ± 0.91 (T)	2.27 ± 0.46 (C) 2.40 ± 1.06 (T)		2.20 ± 0.77 (C) 1.93 ± 0.59 (T)	2.20 ± 0.41 (C) 2.13 ± 0.52 (T)
Asimuddin et al. (2017)	1.15 ± 0.59 (C) 1.25 ± 0.66 (T)	4.19 ± 0.99 (C) 3.61 ± 0.78 (T)	2.55 ± 0.52 (C) 2.45 ± 0.52 (T)	2.55 ± 0.52 (C) 3.09 ± 0.83 (T)		
*CM (C)* vs. *PRF (T)*						
Mehta et al. (2018)	2.84 ± 0.80 (C) 2.44 ± 0.50 (T)	3.00 ± 1.10 (C) 2.80 ± 0.60 (T)				
*PRP (C)* vs. *PRF(T)*						
Bajaj et al. (2013)	3.92 ± 0.93 (C) 4.29 ± 1.04 (T)	2.71 ± 1.04 (C) 2.87 ± 0.85 (T)	2.50 ± 0.83 (C) 2.75 ± 0.94 (T)	1.77 ± 0.52 (C) 1.85 ± 0.49 (T)		
*rhBMP2 (C)* vs. *PRF (T)*						
Sneha et al. (2021)	2.56 ± 1.62 (C) 2.60 ± 0.90 (T)		1.48 ± 1.88 (C) 1.89 ± 1.06 (T)			
**GROUP 3: Therapeutic modalities of PRF with addition of biomaterials/biomolecules**						
*PRF (C)* vs. *PRF + BG (T)*						
**Authors (year)**	**Mean difference in PPD between baseline and final follow‐up (mm)**	**Mean difference in vCAL between baseline and final follow‐up (mm)**	**Mean difference in hCAL between baseline and final follow‐up (mm)**	**Mean difference in vRBF between baseline and final follow‐up (mm)**	**Mean difference in vBF between baseline and final follow‐up (mm)**	**Mean difference in hBF between baseline and final follow‐up (mm)**
Agarwal et al. (2019)	3.80 ± 0.77 (C) 4.00 ± 0.79 (T)	3.55 ± 1.05 (C) 3.90 ± 0.72 (T)			1.60 ± 0.88 (C) 1.90 ± 0.45 (T)	1.40 ± 0.50(C) 1.50 ± 0.76(T)
*PRF(C)* vs. *PRF + AM (T)*						
Kaur and Bathla (2018)	1.33 ± 0.82 (C) 2.53 ± 0.99 (T)	1.33 ± 0.82 (C) 2.53 ± 0.99 (T)	2.20 ± 0.94 (C) 3.00 ± 0.93 (T)			
*PRF(C)* vs. *PRF + metformin (T)*						
Sharma et al. (2017)	1.64 ± 1.76 (C) 3.20 ± 0.88 (T)	2.07 ± 2.08 (C) 2.94 ± 1.07 (T)	0.8 ± 1.23 (C) 1.07 ± 1.16 (T)	0.21 ± 0.45 (C) 0.66 ± 0.84 (T)		
Swami et al. (2021)	3.23 ± 0.90 (C) 3.90 ± 0.78 (T)	2.67 ± 0.88 (C) 3.42 ± 0.93 (T)	1.96 ± 0.80 (C) 2.94 ± 0.80 (T)			
Dhande et al. (2023)	2.96 ± 1.04 (C) 3.81 ± 0.77 (T)	2.57 ± 2.13 (C) 3.60 ± 1.15 (T)	1.98 ± 1.14 (C) 2.77 ± 0.75 (T)			
*PRF(C)* vs. *PRF + bisphosphonates (T)*						
Kanoriya et al. (2017)	3.69 ± 0.76 (C) 4.40 ± 0.57 (T)	3.39 ± 0.49 (C) 4.12 ± 0.60 (T)	2.86 ± 0.06 (C) 3.64 ± 0.90 (T)	2.59 ± 0.32 (C) 2.92 ± 0.25 (T)		
Wanikar et al. (2019)	1.85 ± 0.59 (C) 2.85 ± 0.88 (T)	1.90 ± 0.64 (C) 3.05 ± 0.98 (T)	1.70 ± 0.73 (C) 2.30 ± 0.73 (T)			
*PRF + BG (C)* vs. *PRF + BG + Statins (T)*						
Pradeep et al. (2016)	3.68 ± 1.07 (C) 4.62 ± 1.03 (T)	3.31 ± 0.52 (C) 4.17 ± 0.70 (T)	2.97 ± 0.56 (C) 4.05 ± 0.76 (T)	3.25 ± 0.16 (C) 3.68 ± 0.32 (T)		

Methods for PRF preparation			
Authors (year)	Centrifugation system	Volume of blood drawn	Centrifugation parameters speed (rpm) × time (min)
**GROUP 1: Therapeutic modalities with/without PRF**			
*OFD* vs. *PRF*			
Sharma and Pradeep (2011)	R‐4C (REMI, Mumbai, India)	10 mL	3000 × 10
Bajaj et al. (2013)	R‐4C (REMI, Mumbai, India)	10 mL	400*g* × 10
Siddiqui et al. (2016)	R‐4C (REMI, Mumbai, India)	NR	3000 × 10
Kanoriya et al. (2017)	R‐4C (REMI, Mumbai, India)	10 mL	3000 × 10
Agarwal et al. (2019)	NR	10 mL	400 ***g*** × 12
*BG vs. BG + PRF*			
Lohi et al. (2017)	NR	10 mL	3000 × 10
Rani et al. (2018)	Systonic Lab and Scientific Instruments, India	15 mL	3000 × 12
Basireddy et al. (2019)	R‐8C (REMI, Mumbai, India)	5 mL	3000 × 10
Dambhare et al. (2019)	NR	10 mL	400 ***g*** × 12 min
Serroni et al. (2022)	IntraSpin, Intra‐Lock System Europa SpA, Salerno, Italy	3 × 10 ml	3000 × 10
Nair et al. (2022)	Laboratory centrifuge (Labtech‐Centifuge)	10 mL	700 × 3
**Group 2: Therapeutic Modalities in Comparison to PRF**			
*BG vs. PRF*			
Biswas et al. (2016)	NR	NR	NR
Siddiqui et al. (2016)	R‐4C (REMI, Mumbai, India)	NR	3000 × 10
Asimuddin et al. (2017)	REMI, Mumbai, India	NR	3000 × 10
*CM vs. PRF*			
Mehta et al. (2018)	NR	10 mL	3000 × 10
*PRP* vs. *PRF*			
Bajaj et al. (2013)	R‐4C (REMI, Mumbai, India)	10 mL	400 ***g*** × 10
*rhBMP2 vs. PRF*			
Sneha et al. (2021)	NR	10 mL	2700 × 12
**GROUP 3: Therapeutic modalities of PRF with addition of biomaterials/biomolecules**			
*PRF vs. PRF + BG*			
Agarwal et al. (2019)	NR	10 mL	400 ***g*** × 12
*PRF vs. PRF + AM*			
Kaur and Bathla (2018)	NR	10 mL	2700 × 10
*PRF vs. PRF + metformin*			
Sharma et al. (2017)	R‐4C (REMI, Mumbai, India)	NR	3000 × 10
Swami et al. (2021)	R‐4C (REMI Centrifuge; RCFmax = 603	2 × 10 mL	3000 × 10
Dhande et al. (2023)	Orthophos XG 3D/Ceph, Sirona Dental Systems GmbH, Germany	2 × 10 mL	400 ***g*** × 10
*PRF* vs. *PRF+ bisphosphonates*			
Kanoriya et al. 2017	R‐4C (REMI, Mumbai, India)	10 mL	3000 × 10
Wanikar et al. (2019)	R‐4C (REMI, Mumbai, India)	2 × 10 mL	3000 × 10
*PRF vs. PRF + Statins*			
Pradeep et al. (2016)	R‐4C (REMI, Mumbai, India)	10 mL	3000 × 10

Abbreviations: ♀, female; ALN, alendronate; BF, bone fill; BG, bioactive glass; C, control group; CAL, clinical attachment level; FD, furcation defects; h, horizontal; min = minute; NR, not reported; OFD, open flap debridement; PPD, probing pocket depth; PRF, platelet‐rich fibrin; PRP, platelet‐rich plasma; R, radiographic; RCT, randomized clinical trial; rpm, rotation per minute; *T*, test group, ♂, male; v, vertical.

### 3.3 Interventions and comparisons

A total of 11 categories were divided into three groups as follows:

Group 1: Addition of PRF to a treatment modality
  1. OFD versus PRF^28^, ^29^, ^30^, ^31^, ^32^
  2. BG versus BG + PRF^33^, ^34^, ^35^, ^36^, ^37^, ^38^
Group 2: Comparative studies to PRF
  1. BG versus PRF^30^, ^35^, ^39^, ^40^
  2. CM versus PRF^41^
  3. PRP versus PRF^29^
  4. rhBMP2 versus PRF
Group 3: Addition of biomaterials/biomolecules to PRF
  1. PRF versus PRF + BG^32^
  2. PRF versus PRF + AM^43^
  3. PRF versus PRF + metformin^44^, ^45^, ^46^
  4. PRF versus PRF + bisphosphonate^31^, ^47^
  5. PRF versus PRF + statins^48^

## 4 THERAPEUTIC MODALITIES WITH/WITHOUT PRF (FQ‐1)

For FQ‐1, two comparative subgroups were analyzed (OFD vs. PRF; BG vs. BG + PRF).

### 4.1 Soft tissue parameters

#### 4.1.1 Probing pocket depth reduction

**FIGURE 2:**
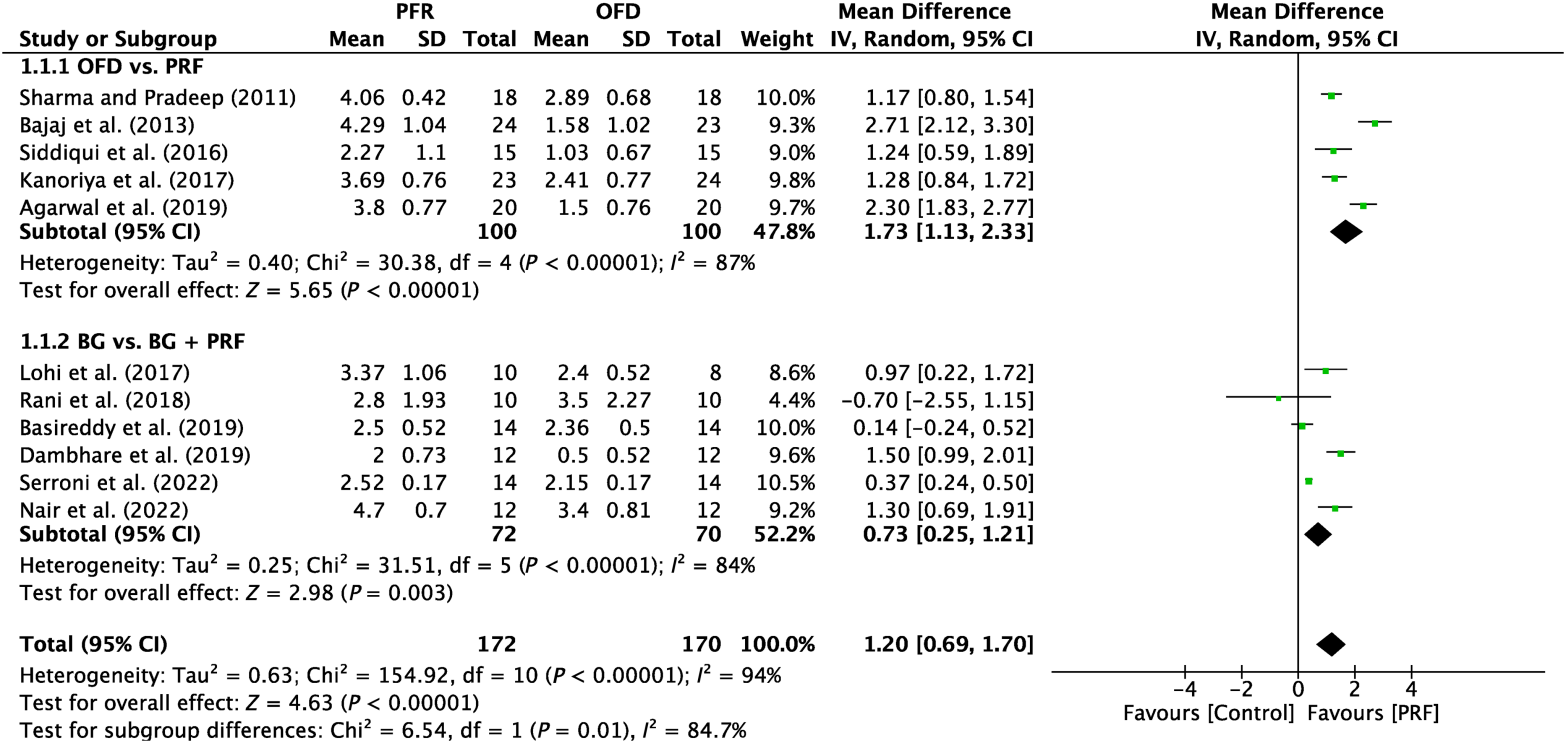
Forest plot for the event reduction in “probing pocket depth” (PPD) (reported in mm) for furcation defects in Group 1: “Therapeutic modalities with/without PRF (FQ‐1).”

A total of 11 studies were analyzed. A high heterogeneity was observed between the studies (*I* ^2^ = 94%; *p* < 0.00001). The subgroups showed a significant impact (*p* < 0.00001; *p* = 0.003) with MD of 1.73 mm (95% CI: 1.13–2.33) and 0.73 mm (95% CI: 0.25–1.21) in favor of the PRF, respectively (Figure 2).

#### 4.1.2 Vertical clinical attachment level gain

**FIGURE 3:**
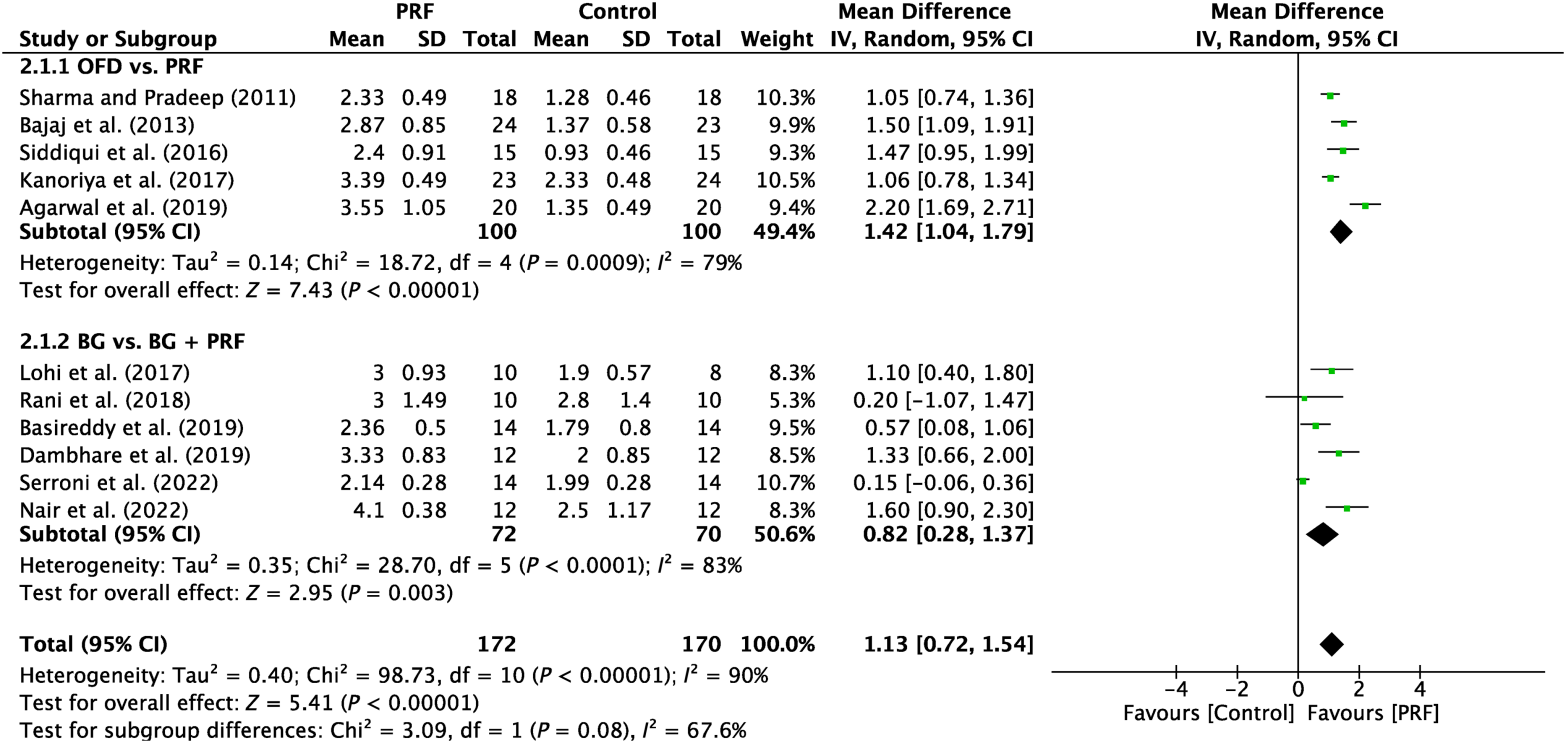
Forest plot for the event “vertical clinical attachment level (VCAL)” (reported in mm) for furcation defects in Group 1: “Therapeutic modalities with/without PRF (FQ‐1).”

A total of 11 studies were analyzed. A high heterogeneity was observed between the studies (*I* ^2^ = 90%; *p* < 0.00001). The subgroups demonstrated a significant effect (*p* < 0.00001; *p* = 0.003) in favor of the PRF, with MD of 1.42 mm (95% CI: 1.04–1.79) and MD of 0.82 mm (95% CI: 0.28–1.37), respectively (Figure 3).

#### 4.1.3 Horizontal clinical attachment level gain

**FIGURE 4:**
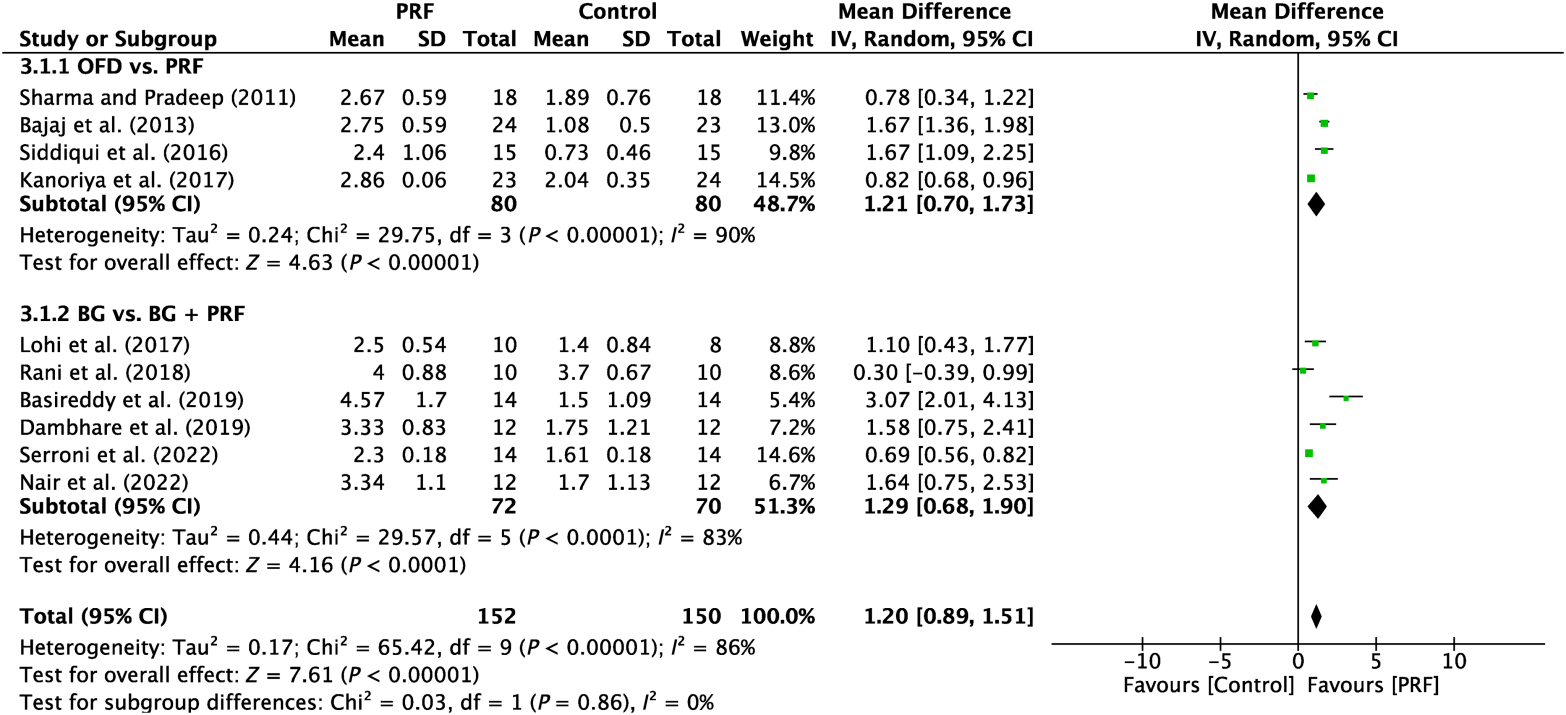
Forest plot for the event “horizontal clinical attachment level (HCAL)” (reported in mm) for furcation defects in Group 1: “Therapeutic modalities with/without PRF (FQ‐1).”

Ten research studies were examined with high variation throughout the research (*I* ^2^ = 86%; *p* < 0.00001). The subgroups demonstrated a significant effect (*p* < 0.00001; *p* < 0.0001) in favor of the PRF, with MD of 1.21 mm (95% CI: 0.70–1.73) and MD of 1.29 mm (95% CI: 0.68–1.90), respectively (Figure 4).

#### 4.1.4 Clinical furcation improvement (complete closure/conversion into class I)

Five studies reported this outcome.^27^, ^32^, ^33^, ^35^, ^36^ In a study by Sharma & Pradeep,^27^ 66.7% of the PRF‐test group saw full closure and 27.7% converted into class I. No information was provided for the OFD group. In four investigations,^32^, ^33^, ^35^, ^36^ only one trial demonstrated full furcation closure when comparing BG versus BG + PRF^35^ at a frequency of 16.7% (BG) compared to 50% (BG + PRF). Regarding the conversion of class I, two studies^33^, ^35^ found no difference, while two other studies^32^, ^36^ reported a higher frequency for the test group (Table 2).

**TABLE 2:** Furcation closure/conversion (class II to class I).

Study (year)	Treatment arms	Furcation closure, *n* (%)	Furcation conversion, *n* (%)
**GROUP 1: Therapeutic modalities with/without PRF**			
*OFD* vs. *PRF*			
Sharma and Pradeep (2011)^27^	C: OFD T: OFD + PRF	C: N.R. T: 12 (66.7%)	C: N.R. T: 5 (27.7%) to class I
Bajaj et al. (2013)^28^	OFD OFD + PRF	N.R.	N.R.
Siddiqui et al. (2016)^29^	OFD OFD + PRF	N.R.	N.R.
Kanoriya et al. (2017)^30^	C: OFD T: OFD + PRF	N.R.	N.R.
Agarwal et al. (2019)^31^	C: OFD T: OFD + PRF	N.R.	N.R.
*BG vs. BG + PRF*			
Lohi et al. (2017)^32^	OFD + BG OFD + BG + PRF	C: 0 T: 0	C: 6 (60%) to class I T: 8 (100%) to class I
Rani et al. (2018)^33^	C: OFD + BTCP T: OFD + BTCP + PRF	C: 0 T: 0	C: 9 (90%) to class I T: 9 (90%) to class I
Basireddy et al. (2019)^34^	OFD + DFDBA OFD + DFDBA + PRF	N.R.	N.R.
Dambhare et al. (2019)^35^	C: OFD + HA and β‐TCP T: OFD + HA and β‐TCP + PRF	C: 2 (16.66%) T: 6 (50%)	C: 6 (50%) to class I T: 6 (50%) to class I
Serroni et al. (2022)^36^	C: OFD + AB T: OFD + AB + PRF	C: 0 T: 0	C: 11 (61.1%) to class I T: 12 (66.6%) to class I
Nair et al. (2022)^37^	OFD + nano‐HA OFD + nano‐HA + i‐PRF	N.R.	N.R.
**Group 2: Therapeutic modalities in comparison to PRF**			
*BG vs. PRF*			
Biswas et al. (2016)^38^	OFD + BG OFD + PRF	N.R.	N.R.
Siddiqui et al. (2016)^29^	OFD + BG OFD + PRF	N.R.	N.R.
Asimuddin et al. (2017)^39^	C: OFD + DFDBA + CM T: OFD + PRF	N.R.	N.R.
*CM vs. PRF*			
Mehta et al. (2018)^40^	C: OFD + DFDBA + CM T: OFD + DFDBA + PRF	N.R.	N.R.
Bajaj et al. (2013)^28^	OFD + PRP OFD + PRF	N.R.	N.R.
*rhBMP2 vs. PRF*			
Sneha et al. (2021)^41^	C: OFD + collagen sponge + rhBMP2 T: OFD + PRF	N.R.	N.R.
**GROUP 3: Therapeutic modalities of PRF with addition of biomaterials/biomolecules**			
*PRF vs. PRF + BG*			
Agarwal et al. (2019)^31^	C: OFD + PRF T: OFD + PRF + DFDBA	N.R.	N.R.
*PRF vs. PRF + AM*			
Kaur and Bathla (2018)^42^	C: OFD + PRF T: OFD + PRF + AM	N.R.	N.R.
*PRF vs. PRF + metformin*			
Sharma et al. (2017)^43^	C: OFD + PRF T: OFD + PRF + MF	N.R.	N.R.
Swami et al. (2022)^44^	C: OFD + PRF T: OFD + PRF + MF	C: 11 (52%) T: 16 (76%)	C: 10 (47%) to class I T: 5 (23%) to class I
Dhande et al. (2023)^45^	C: OFD + PRF T: OFD + PRF + MF	N.R.	N.R.
*PRF* vs. *PRF+ bisphosphonates*			
Kanoriya et al. (2017)^30^	C: OFD + PRF T2: OFD + PRF + 1% ALN	N.R.	N.R.
Wanikar et al. (2019)^46^	C: OFD + PRF T: OFD + PRF + 1%ALN	N.R.	N.R.
*PRF vs. PRF + statins*			
Pradeep et al. (2016)^47^	C0: OFD C: OFD + HA + PRF T: OFD + HA PRF + 1.2% RSV	N.R.	N.R.

Abbreviations: AB, autogenous bone; AM, Amniotic membrane; ALN, alendronate; BG, bone graft; BTCP, beta tricalcium phosphate; CM, collagen membrane; DFDBA, demineralized freeze‐dried bone allograft; HA, hydroxyapatite; i‐PRF, injectable platelet rich fibrin; MT, metformin; N.R., not reported; OFD, open flap debridement; PRF, platelet rich fibrin; PRP, platelet rich plasma; rhBMP2, recombinant human bone morphogenetic protein 2; RSV, rosuvastatin.

### Hard tissue parameters

Because there are so few studies examining VBL, HBL, or RBF, no meta‐analysis was done when looking at these parameters. However, three studies showed that the PRF group had a considerable advantage when comparing VBF gain when comparing OFD to OFD/PRF.^28^, ^29^, ^31^ Improved VBL and HBL gain were observed in two different investigations (Table 1).^30^, ^32^ When comparing BG to BG/PRF, no significant differences were seen across the groups, except for research conducted by Lohi et al.^33^

## 5 THERAPEUTIC MODALITIES IN COMPARISON TO PRF (FQ‐2)

For FQ‐2, four comparative subgroups were analyzed (BG vs. PRF; CM vs. PRF; PRP vs. PRF; rhBMP2 vs. PRF).

### 5.1 Soft tissue parameters

#### 5.1.1 Probing pocket depth reduction

**FIGURE 5:**
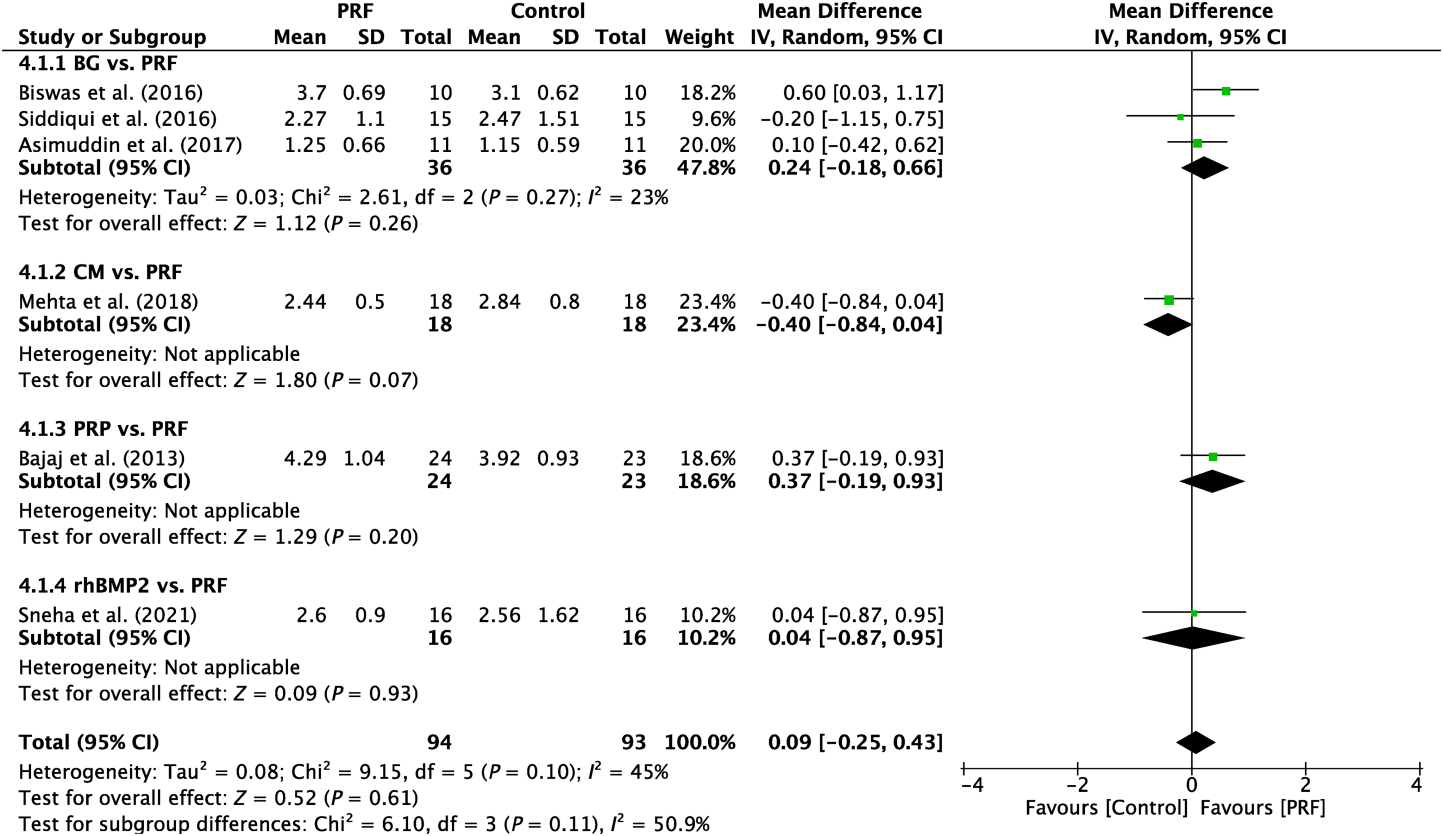
Forest plot for the event reduction in “probing pocket depth” (PPD) (reported in mm) for furcation defects in Group 2: “Therapeutic modalities in comparison to PRF (FQ‐2).”

Six studies were examined in total reporting a modest variation across the studies (*I* ^2^ = 45%; *p* < 0.10). No significant differences were seen in between the subgroups analyzed (*p* = 0.26; *p* = 0.07; *p* = 0.20; *p* = 0.93; Figure 5).

#### 5.1.2 Vertical clinical attachment level gain

**FIGURE 6:**
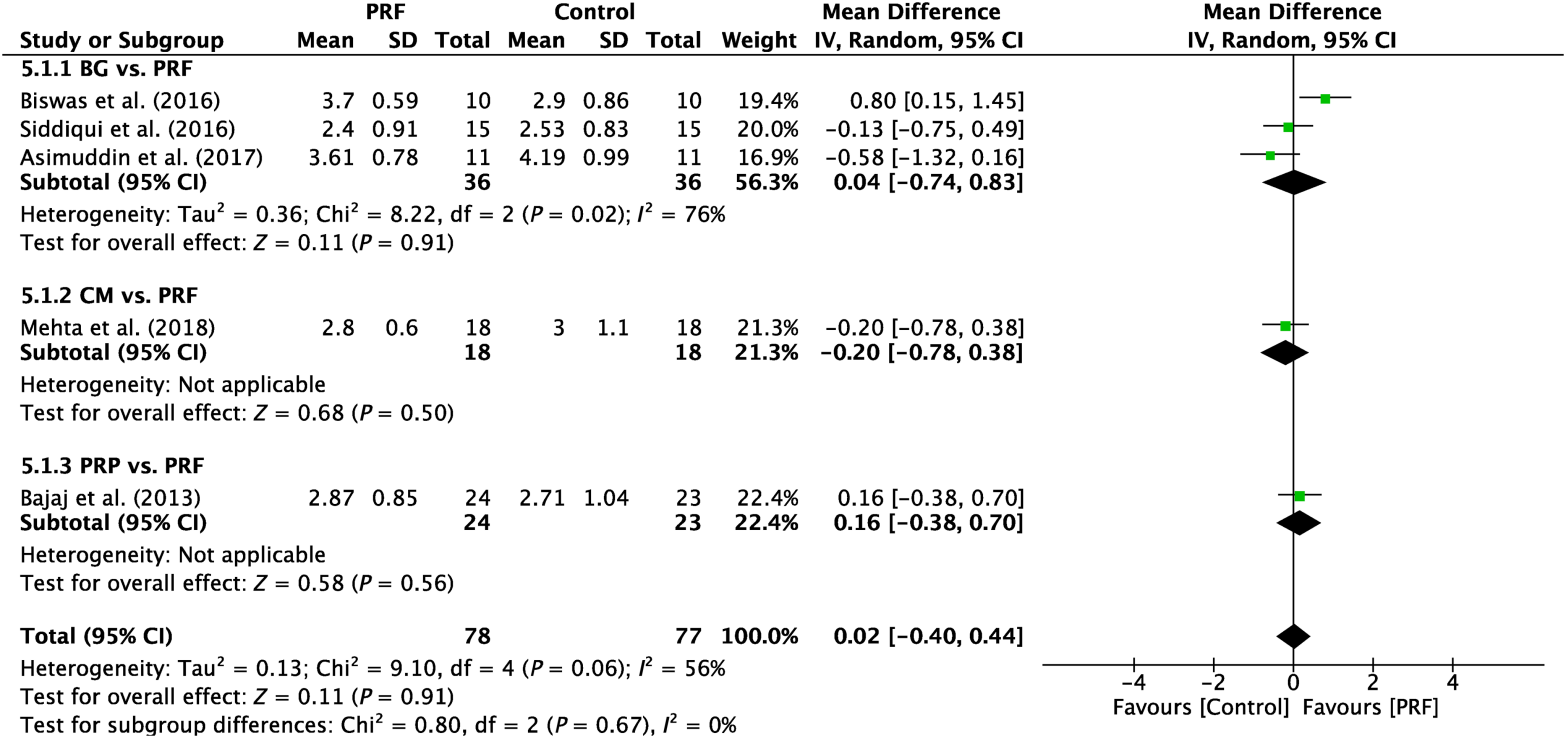
Forest plot for the event “vertical clinical attachment level (VCAL)” (reported in mm) for furcation defects in Group 2: (FQ‐2).”

Five studies were examined in total. Significant variability was identified across the studies (*I* ^2^ = 56%; *p* = 0.06). No significant differences were seen between the subgroups analyzed (*p* = 0.91; *p* = 0.50; *p* = 0.56; Figure 6).

#### 5.1.3 Horizontal clinical attachment level gain

**FIGURE 7:**
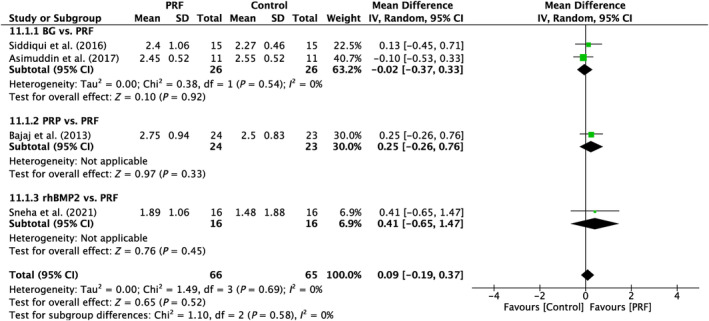
Forest plot for the event “horizontal clinical attachment level (HCAL)” (reported in mm) for furcation defects in Group 2: “Therapeutic modalities in comparison to PRF (FQ‐2).”

Analysis was done on four trials in total. There was no discernible variability across the studies (*I* ^2^ = 0%; *p* = 0.69). No significant differences were seen between the subgroups analyzed (*p* = 0.92; *p* = 0.33; *p* = 0.45; Figure 7).

### 5.2 Hard tissue parameters

Again, due to the variation in reported results for each parameter, no meta‐analysis could be carried out when examining VBL, HBL, or RBF. However, research by Asimuddin et al.^40^ and Bajaj et al.^29^ discovered no significant variations in RBF between the test and control groups. An investigation by Biswas et al.^39^ reported no significant differences in the average HBF values and Siddiqui et al.^30^ also found no differences between HBF and VBF when comparing OFD/BG versus OFD/PRF.

## 6 THERAPEUTIC MODALITIES OF PRF WITH ADDITION OF BIOMATERIALS/BIOMOLECULES (FQ‐3)

For FQ‐3, five comparative subgroups were analyzed (PRF vs. PRF + BG; PRF vs. PRF + AM; PRF vs. PRF + metformin; PRF vs. PRF + bisphosphonates; PRF vs. PRF + statins).

### 6.1 Soft tissue parameters

#### 6.1.1 Probing pocket depth reduction

**FIGURE 8:**
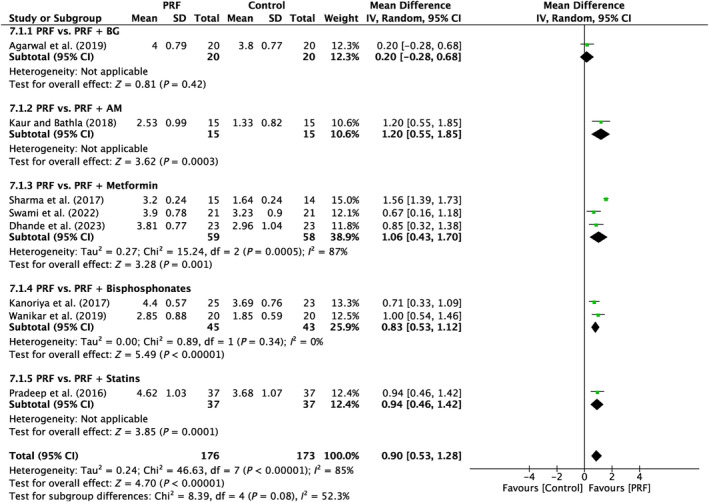
Forest plot for the event reduction in “probing pocket depth” (PPD) (reported in mm) for furcation defects in Group 3: “Therapeutic modalities using PRF with addition of biomaterials/biomolecules (FQ‐3).”

Analysis was done on eight papers in total. There was a high amount of variation throughout the research (*I* ^2^ = 85%; *p* < 0.00001). Four subgroups (PRF vs. PRF + AM; PRF vs. PRF + metformin; PRF vs. PRF + bisphosphonates; PRF vs. PRF + statins) demonstrated a significant difference (*P* = 0.0003; *P* = 0.001; *P* < 0.00001; *P* = 0.0001) in favor of PRF, with MD of 1.2 mm (95% CI:0.55–1.85), 1.06 mm (95% CI: 0.43–1.70), 0.83 mm (95% CI:0.53–1.12), 0.94 mm (95% CI:0.46–1.42). One subgroup (PRF vs. PRF + BG) did not demonstrate a significant difference (*p* = 0.42) with MD of 0.20 mm (95% CI:–0.28–0.68). (Figure 8).

#### 6.1.2 Vertical clinical attachment level gain

**FIGURE 9:**
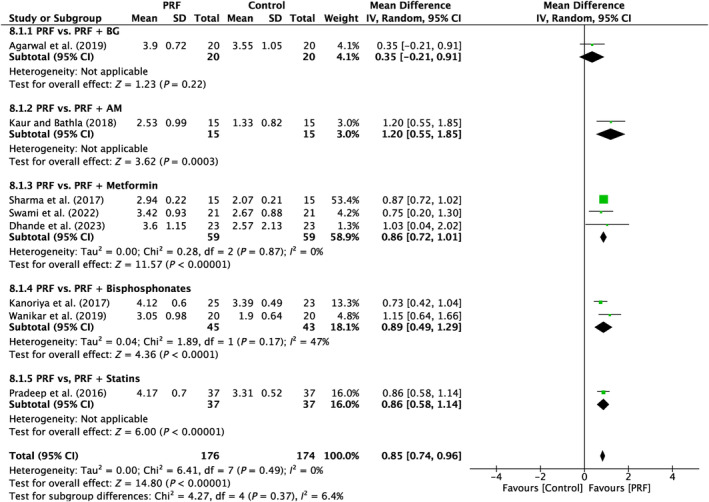
Forest plot for the event “vertical clinical attachment level (VCAL)” (reported in mm) for furcation defects in Group 3: “Therapeutic modalities using PRF with addition of biomaterials/biomolecules (FQ‐3).”

Eight studies were examined in total. There was a lack of variability reported across the studies (*I* ^2^ = 0%; *p* = 0.49). Five subgroups (PRF vs. PRF + BG; PRF vs. PRF + AM; PRF vs. PRF + metformin; PRF vs. PRF + bisphosphonates; PRF vs. PRF + statins) demonstrated a significant difference (*p* = 0.003; *p* < 0.00001; *p* < 0.0001; *p* < 0.00001) in favor of the PRF, with MD of 1.2 mm (95% CI: 0.55–1.85), 0.86 mm (95% CI: 0.72–1.01), 0.89 mm (95% CI: 0.49–1.29), 0.86 mm (95% CI: 0.58–1.14), respectively (Figure 9).

#### 6.1.3 Horizontal clinical attachment level gain

**FIGURE 10:**
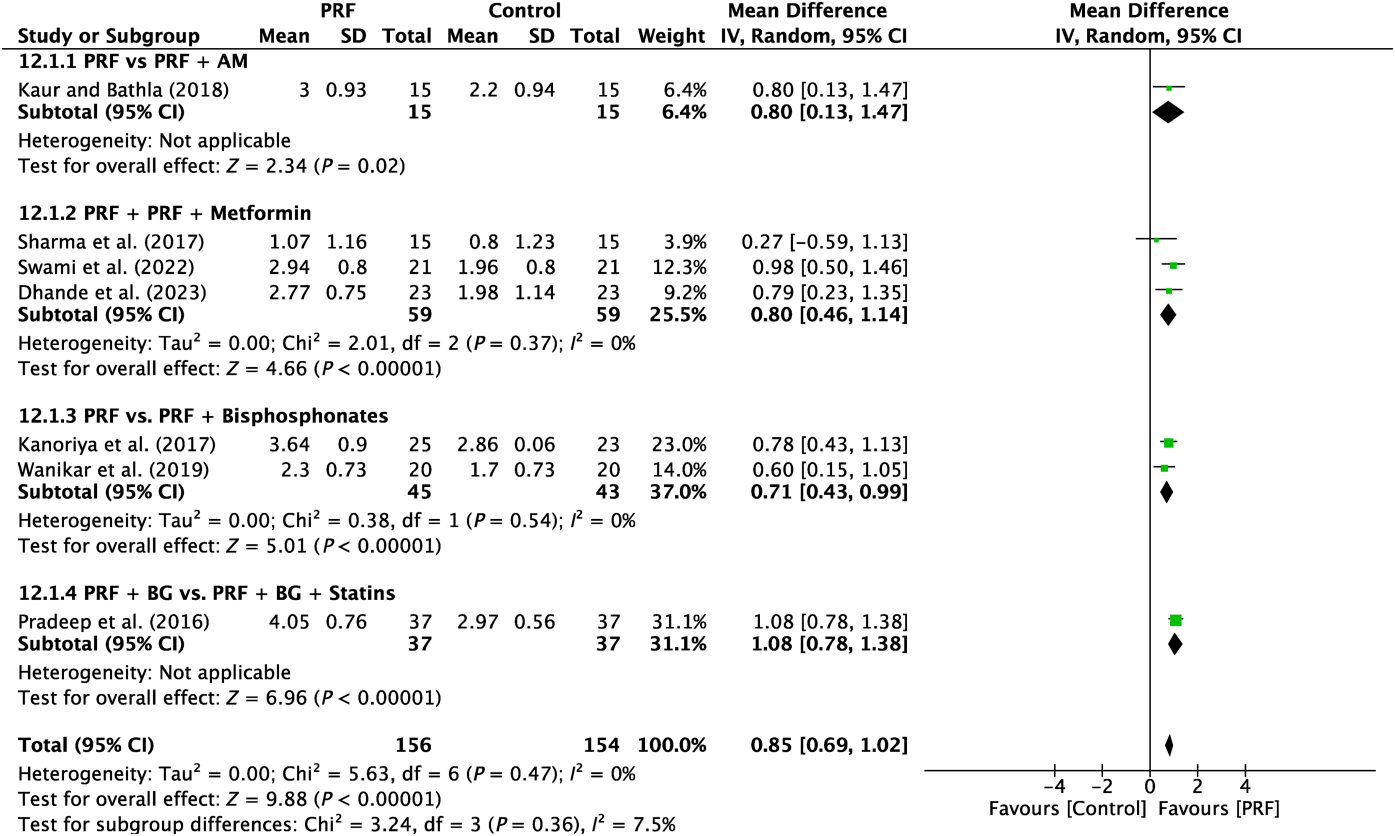
Forest plot for the event “horizontal clinical attachment level (HCAL)” (reported in mm) for furcation defects in Group 3: “Therapeutic modalities using PRF with the addition of biomaterials/biomolecules (FQ‐3).”

Seven studies were examined in total. There was a lack of variability reported across the studies (*I* ^2^ = 0%; *p* = 0.47). Four subgroups (PRF vs. PRF + AM; PRF vs. PRF + metformin; PRF vs. PRF + bisphosphonates; PRF vs. PRF + statins) demonstrated a significant difference (*p* = 0.02; *p* < 0.00001; *p* < 0.00001; *p* < 0.00001) in favor of the PRF, with MD of 0.8 mm (95% CI: 0.13–1.47), 0.80 mm (95% CI: 0.46–1.14), 0.71 mm (95% CI: 0.43–0.99), 1.08 mm (95% CI: 0.78–1.38), respectively (Figure 10).

#### 6.1.4 Clinical furcation improvement (complete closure/conversion into class I)

This result was shown in research that compared the effects of PRF alone against PRF combined with metformin.^44^ For both class I conversion and control versus test, the frequency of full closure was 47% versus 23% and 52 versus 76%, respectively (Table 2).

### 6.2 Hard tissue parameters

Since each of the analyzed groups only reported RBF, VBF, or HBF in a single study, no meta‐analysis could be done for FQ‐3. Overall, the patterns for hard tissue metrics mirrored PPD decrease and CAL increases for each of the recognized groups, as noted in Table 1.

#### 6.2.1 Overall risk of bias

One study^37^ was assessed at low risk of bias in all five domains, three trials were with high risk of bias in at least one domain^33^, ^38^, ^42^ and the remaining 17 studies of the included 21 studies were with “some concerns” in at least one domain. Only the missing information on allocation concealment was a factor in the assessment of the unknown risk of bias (some concerns) among those 17 studies. These 10 studies would also have had an overall low risk of bias if this had been made explicit in the articles' descriptions of the randomization technique (Appendix S1).

#### 6.2.2 Randomization process

Nineteen trials described the method of randomization. In two trials, the method of randomization was uncertain^33^ or not stated.^42^ Seven used a computer table.^29^, ^31^, ^32^, ^37^, ^45^, ^46^, ^48^ In 11 trials, the method of randomization was a coin toss or lottery method.^28^, ^34^, ^35^, ^36^, ^38^, ^39^, ^40^, ^41^, ^43^, ^44^, ^47^ In four studies, the allocation concealment was secured by sealed envelopes.^30^, ^37^, ^41^, ^43^

#### 6.2.3 Deviations from the intended interventions

Since the therapist could not be blinded to the surgical operation, we were unable to rate the operator's performance bias. For this domain, there was a low risk of bias in the 17 studies. Two studies were found to have an unknown risk of bias (some concerns) due to no power estimates or disclosure of the dropout rate.^39^, ^43^ Two studies exhibited a significant dropout rate (>22%) resulting in a high risk of bias.^41^, ^42^

#### 6.2.4 Missing outcome data

For this domain, there was little risk of bias in the 17 trials. Two were with some concerns.^42^, ^43^ and two had a high risk of bias due to a lack of knowledge on the causes of dropout.^33^, ^38^

#### 6.2.5 Measurement of the outcome

Fifteen studies exhibited a minimal risk of bias, whereas the remaining six studies raised some concerns owing to the absence of information about the blinding of outcome assessors.^30^, ^38^, ^39^, ^40^, ^44^, ^46^

#### 6.2.6 Selection of the reported result

There were 19 studies identified that had a low risk of bias in this specific area, whereas two trials had some concerns about bias.^33^, ^38^ The figure of ROB 2 is included in Appendix S1.

## 7 DISCUSSION

The present SR and meta‐analysis investigated the use of PRF for reconstructive surgery in furcation defects as evaluated in RCTs compared to all other treatment modalities. The aim was to address the use and recommendations more specifically for PRF for the treatment of periodontal class II furcation defects. Overall, the majority of studies to date compared the use of OFD/PRF versus OFD alone or OFD/BG versus OFD/BG/PRF (Table 1). Furthermore, additional comparative studies have investigated OFD/PRF versus many commonly utilized regenerative modalities including OFD/BG, OFD/CM, OFD/PRP, OFD/rhBMP2. A final third group of studies compared the standard use of OFD/PRF with addition of a biomaterial or biomolecule such as BG, metformin, bisphosphonates, and statins. Next, we highlight and discuss the summary of evidence from the current categories and further discuss the strengths and limitations of each comparative analysis.

### 7.1 GROUP 1: Therapeutic Modalities with/without PRF

#### 7.1.1 OFD alone versus with PRF

In all, five trials assessed the efficacy of PRF in addition to OFD as opposed to OFD alone (Table 1).^28^, ^29^, ^30^, ^31^, ^32^ Overall, statistically significant clinical benefits in mean PD reduction were seen in all five investigations (Figure 2), as well as mean HCAL and VCAL gain (Figures 3 and 4). In conclusion, it was shown that, on average, the outcomes from five RCTs showed a statistically significant relative PPD decrease of about 1.3 mm and a CAL gain of about 1.5 mm when PRF was added to intrabony defects after OFD (Table 1).

#### 7.1.2 Bone graft versus bone graft + PRF

In a third series of investigated studies, six studies evaluated the additional use of PRF to BG when compared to BG alone (Table 1).^33^, ^34^, ^35^, ^37^, ^38^ Of the six studies, four demonstrated a statistically significant improvement in PPD and HCAL/VCAL gain when compared to BG alone,^33^, ^34^, ^37^, ^38^ while the other two studies demonstrated no statistically significant difference (Figure 2). The study by Basireddy et al. demonstrated no additional benefits when PRF was added to DFDBA aside from the clinical observation that PRF seemed to have improved soft tissue healing.^35^ Furthermore the study by Rani et al. showed no improvements either.^34^ Potential reasons for variability in the findings could be due to the bone grating material selected. For instances, PRF may prove to be more beneficial in BGs that do not contain growth factors such as xenografts or synthetic alloplasts, whereas allografts already contain a number of regenerative growth factors contained within their scaffold. Nevertheless, when observing data from intrabony defect regeneration, the combination of DFDBA + PRF has led to significant clinical advantages across many studies when compared to DFDBA alone.^49^, ^50^, ^51^ Since data are limited in furcation defect regeneration with PRF when combined with BGs comparatively, it would be valuable to have more studies conducted comparing various bone grafting materials in combination with PRF. Nevertheless, all studies demonstrated improvements in soft tissue wound healing. Another factor that may be relevant to current discussion is the fact that PRF also includes supraphysiological concentrations of leukocytes that may further reduce and defend against potential bacterial invasion/contamination. Basic science studies have now demonstrated that PRF possesses antibacterial as well as anti‐inflammatory properties.^52^, ^53^, ^54^ Recent research has shown that PRF has the ability to favor M2 macrophage polarization and also decreases tissue inflammation.^52^, ^53^ It also possesses some antibacterial/antimicrobial activity, thereby favoring potential wound healing of periodontal pockets.^55^, ^56^ Taken together, each of the abovementioned parameters is thought to at least in part contribute toward periodontal regeneration when PRF is utilized in combination with a BG.

### 7.2 Group 2: therapeutic modalities in comparison to PRF

#### 7.2.1 Bone graft versus PRF

In a second group of studies, studies compared the use of PRF versus other biomaterials for the treatment of furcation defects (Table 1). The most common comparison was that between OFD/BG versus OFD/PRF.^30^, ^39^, ^40^ In general, little statistically significant difference was found between both groups. One study reported statistically significantly better results for PRF with respect to soft tissue healing.^39^ Overall the meta‐analysis demonstrated no statistically significant differences in PPD reduction, HCAL gain, or VCAL gain between the two groups, though few studies were investigated. Therefore, additional RCTs are required to fully better address this comparison.

#### 7.2.2 PRF versus CM, PRP, or rhBMP2

Three additional studies compared OFD/CM versus OFD/PRF,^41^ OFD/PRP versus OFD/PRF,^29^ and OFD/rhBMP2 versus OFD/PRF^42^ (Table 1). Each of the studies reported no advantages of one group over the other. When these studies are compared to those reported previously for intrabony defects, the comparison between PRF versus a collagen barrier membrane yielded no statistically significant difference in terms of PD reduction, but PRF demonstrated statistically significant improvements for CAL and RBF favoring the PRF group.^57^ Another study by Pham et al.^58^ demonstrated comparable results when two and three wall intrabony defects were treated with either OFD/BM or OFD/PRF. Two studies previously investigating PRP versus PRF for intrabony defect regeneration also showed no differences between groups.^59^, ^60^ In the rhBMP2 group, a significant increase in bone fill was reported, though the results were minimal. It must be noted that several authors have however not generally recommended the use of rhBMP2 for periodontal regeneration of either intrabony versus furcation defects mainly owing to the chance of ankylosis.^61^, ^62^

### 7.3 Group 3: Therapeutic modalities of PRF with addition of biomaterials/biomolecules

#### 7.3.1 Addition of a biomaterial to PRF (PRF vs. PRF/BG; PRF vs. PRF/AM)

Only one study compared the additional use of a bone graft to PRF.^32^ In general, it was reported that the additional use of a BG primarily improved bone fill. In another study, OFD/PRF was investigated when an amniotic membrane was added.^43^ The additional use of an AM benefited all investigated parameters including PPD reduction, CAL gain, and bone fill.^43^

#### 7.3.2 Addition of a biomolecule to PRF (metformin, bisphosphonates, statins)

Interestingly, one of the largest study groups investigated the additional use of small biomolecules to OFD/PRF. These included a total of six studies whereby three studies investigated the combination of PRF with metformin,^44^, ^45^, ^46^ two studies investigated the combination of PRF with bisphosphonate,^31^, ^47^ and one study investigated the combination of PRF with statins.^48^ Overall, each study resulted in clinical benefits of additionally adding a small biomolecule and this has also been reported to lead to significantly better clinical outcomes when small biomolecules were additionally added to PRF for the treatment of intrabony defects.^63^, ^64^, ^65^, ^66^, ^67^

Although little research has been conducted to evaluate their potential advantages, these relatively new discoveries provide support for the current inclination toward personalized medicine as regenerative approaches. Hence, future studies focusing on specific patient groups, such as women with osteoporosis, could explore the localized administration of supplementary biomolecules, like bisphosphonates, to enhance targeted bioactivity, specifically antiresorptive properties. This approach would promote a more individualized treatment protocol. Moreover, the use of antibiotic treatment in certain individuals with severe periodontitis may potentially gain advantages from a more individualized approach to antibiotic therapy. PRF may be used as a three‐dimensional matrix to transport tiny biomolecules over a long period of time. This makes PRF a potential method for delivering therapeutic drugs, as previously documented^68^ in studies and more recently in clinical trials in the field of periodontology.^69^, ^70^, ^71^ However, the mechanisms by which some tactics, such as combining antibiotics, work are still relatively unknown. It is also uncertain if these techniques have any negative effects on the cells or growth factors produced by PRF. Further clinical benefits may be achieved by future basic science research that explores the potential of PRF as a drug delivery system for diverse local therapeutic agents and biomolecules, with a focus on enhancing our knowledge of this technology. The aforementioned strategies are only documented in individual RCTs, necessitating much more study on the subject.

### 7.4 Implications for clinical practice and future direction

Although the use of PRF in ordinary clinical practice for treating furcation defects is still relatively new, it is worth mentioning that 21 RCTs have examined its potential for periodontal regeneration/repair in the last 15 years. Noteworthy, however, a very high heterogeneity was found in the investigated studies. The presence of a blood clot is an essential need for periodontal regeneration to occur, provided that all bacterial infections have been fully eradicated. Existing research indicates that blood clot formation alone may effectively fill certain intrabony defects, particularly those where space preservation is not as significant a concern as it would theoretically be in furcation defects.^72^ Consequently, the use of combination techniques, including bone grafting materials, seems to be the preferred therapeutic choice. However, there is a lack of clinical recommendations regarding the appropriate use of each methodology in this field. Additionally, recent guidelines by the European Federation of Periodontology have recommended a follow‐up time of 12 months. The present systematic review had a mean follow‐up period of 9.42 months across all studies. A recommendation to provide data at a minimum 12‐month follow‐up for furcation defect RCTs is recommended to better evaluate the regenerative/healing potential of PRF across such studies.

There are many research areas that still need to be prioritized in this field. It is worth noting that there has been no comprehensive investigation that has examined or described the therapeutic advantages of employing PRF for periodontal regeneration at the histology level in a well‐defined human study. Existing research has firmly proven that PRF has a preference for promoting the healing of soft tissue wounds over hard tissues.^73^ In order to fully understand the regenerating capabilities of the tissues affected by periodontitis, namely the periodontal ligament (PDL), cementum, and alveolar bone, it is necessary to conduct histological evaluations. Ideally, these evaluations should be performed in human research.

Another limitation is in the documented variations in the preparation of PRF. Indeed, most studies utilized a relatively high centrifugation speed of 3000 rpm for a duration of 10 min. However, many of these studies have not yet investigated the effects of RCF in relation to the size and radius of the centrifuge. Several position papers have been produced on this issue to emphasize the need to include PRF protocols reporting RCF in publications in order to enhance reproducibility.^74^, ^75^ Multiple studies have shown that changes in PRF production, such as decreasing RCF values during the spin cycle,^76^, ^77^ using horizontal centrifugation to produce PRF,^78^, ^79^ and selecting specific PRF tubes^80^ can significantly influence the quality of the final PRF biomaterial. These modifications contribute to the improved optimization of the technology.^81^

To summarize, all therapy methods that included PRF in their surgical approach (Group 1) showed superior results in terms of improving clinical characteristics of class II furcation defects. Each treatment modality that compared PRF alone to other regenerative techniques (Group 2) saw comparable clinical results in both groups. The treatment approaches that use PRF, together with the incorporation of specific biomaterials or biomolecules (Group 3), have shown enhanced clinical results, particularly when tiny biomolecules such as metformin, bisphosphonates, and statins were included. Further investigation is required owing to the very high heterogeneity found in the investigated studies to determine the optimal circumstances for using PRF in combination with regenerative biomaterials instead of using it as the sole “graft” material.

Many studies analyzed in this review presented methodological variation (e.g., settings, sample size, and follow‐up time). Because of this, all meta‐analyses were evaluated using the random effect model and the results should be interpreted with caution. New trials with greater method standardization are fundamental in the future.

## 8 CONCLUSION

The data from this SR demonstrate that the use of PRF associated with OFD improves the VCAL/HCAL and RFB parameters for the treatment of class II furcation defects when compared to the isolated use of OFD. Additionally, combining BG and PRF can lead to statistically significant improvements in VCAL/HCAL. Future research may be warranted to evaluate the use of PRF in combination with various additional small biomolecules such as metformin, bisphosphonates, statins and/or antibiotics to additionally improve clinical outcomes in complex defects. In addition, animal and human histological evidence are needed to verify if PRF actually leads to true periodontal regeneration. More RCTs with a mean follow‐up of 12 months is recommended in future studies. The results of this review must be interpreted with caution due to the methodological variation presented by the included studies.

## Supporting information


Appendix S1.

## Data Availability

Data sharing not applicable to this article as no data sets were generated or analyzed during the current study.
