# Supplementary material for: Periodontal regeneration using platelet‐rich fibrin. Furcation defects: A systematic review with meta‐analysis
Source: Periodontol 2000. 2024 Sep 26;97(1):191–214. doi: 10.1111/prd.12583 (PMC11808472; doi:10.1111/prd.12583)
Supplement: Supplementary file 1 — Appendix S1. [file PRD-97-191-s001.pdf]

Unique ID	Study ID	Weight	D1	D2	D3	D4	D5	Overall
Agarwal 2019	Furcations	1	!	+	+	+	+	!
Asimuddin 2017	Furcations	1	!	+	+	!	+	!
Bajaj 2013	Furcations	1	!	+	+	+	+	!
Basireddy 2019	Furcations	1	!	+	+	+	+	!
Biswas 2016	Furcations	1	!	!	+	!	+	!
Dambhare 2019	Furcations	1	!	+	+	+	+	!
Dhande 2023	Furcations	1	!	+	+	!	+	!
Kanoriya 2017	Furcations	1	!	+	+	+	+	!
Kaur 2018	Furcations	1	+	!	+	+	+	!
Lohi 2017	Furcations	1	-	+	-	+	!	-
Mehta 2018	Furcations	1	+	+	!	+	+	!
Nair 2022	Furcations	1	!	-	-	!	!	-
Pradeep 2016	Furcations	1	!	+	+	+	+	!
Rani 2018	Furcations	1	!	+	+	+	+	!
Serroni 2022	Furcations	1	+	+	+	+	+	+
Sharma 2011	Furcations	1	!	+	+	+	+	!
Sharma 2017	Furcations	1	!	+	+	!	+	!
Siddiqui 2016	Furcations	1	+	+	+	!	+	!
Sneha 2021	Furcations	1	!	-	!	+	+	-
Swami 2022	Furcations	1	!	+	+	+	+	!
Wanikar 2019	Furcations	1	!	+	+	+	+	!

+ Low risk
! Some concerns
- High risk

D1 Randomisation process
D2 Deviations from the intended interventions
D3 Missing outcome data
D4 Measurement of the outcome
D5 Selection of the reported result
